# Prognostic relevance of TTF-1 expression in stage I adenocarcinoma

**DOI:** 10.18632/oncotarget.22489

**Published:** 2017-11-18

**Authors:** Chao Zhou, Jikai Zhao, Jinchen Shao, Wentao Li

**Affiliations:** ^1^ Department of Thoracic Surgery, Shanghai Chest Hospital, Shanghai Jiao Tong University, Shanghai 200030, China; ^2^ Department of Pathology, Shanghai Chest Hospital, Shanghai Jiao Tong University, Shanghai 200030, China

**Keywords:** thyroid transcription factor-1, lung adenocarcinoma, subtype, adjuvant chemotherapy, prognosis

## Abstract

Tyroid transcription factor-1 (TTF-1) motivates the differentiation and development of bronchioloalveolar cells. The association of TTF-1 expression with prognosis in stage I adenocarcinoma is unclear. This study enrolled patients with resected stage I pulmonary adenocarcinoma who had TTF-1 immunostaining. All the corresponding clinicopathologic data including sex, age, smoking history, pathologic T stage, pathologic disease stage, surgical procedure, subtypes, follow-up records and adjuvant chemotherapy were investigated. Totally, 126 adenocarcinomas with TTF-1− and 2687 adenocarcinomas with TTF-1+ were subjected to the study. Among adenocarcinomas with TTF-1−, the major subtype was acinar-predominant adenocarcinomas, followed by invasive mucinous and papillary subtypes while acinar, papillary and minimally invasive adenocarcinoma were in the majority among adenocarcinomas with TTF-1+. The status of TTF-1 expression was not a significant factor for relapse-free survival (RFS) and overall survival (OS). Furthermore, there was no survival difference between the two groups (RFS: *p* = 0.2474; OS: *p* = 0.1480). When confined to stage IB adenocarcinomas with TTF-1−, whether received adjuvant chemotherapy made no difference to RFS and OS (RFS: *p* = 0.2707; OS: *p* = 1.000), as was the case in stage IB adenocarcinomas with TTF-1+ (RFS: *p* = 0.9161; OS: *p* = 0.1100). Within follow-up period, there was significant difference in post-recurrence survival (PRS) for TTF-1− patients compared with those TTF-1+ patients (Log-rank *p* = 0.0113). However, regarding to the recurrence site, there was no difference between TTF-1− patients and TTF-1+ patients in patients with stage I adenocarcinoma (*p* = 0.771) In conclusion, there is no significant difference in RFS and OS between TTF-1− group and TTF-1+ group, but TTF-1 negative adenocarcinoma has significantly worse PFS in patients with stage I adenocarcinoma. Moreover, chemotherapeutic efficacy between TTF-1+ and TTF-1− stage IB adenocarcinomas did not differ.

## INTRODUCTION

Lung cancer remains the leading cause of cancer death worldwide in recent years [1]. Lung adenocarcinoma, accounting for nearly 50% of all lung cancer, is the most common histologic subtype and the morbidity rate is increasing year by year [2]. Prognosis of lung adenocarcinoma has raised concern by the International Association for the Study of Lung Cancer (IASLC)/American Thoracic Society (ATS)/European Respiratory Society (ERS) in 2011 [3].

Thyroid transcription factor-1 (TTF-1), known as a member of homeodomain-containing nuclear transcriptional protein of the Nkx2 gene family, motivates the differentiation and development of bronchioloalveolar cells [4]. In normal adult lung, the expression of TTF-1 is confined mainly to terminal respiratory units [5, 6]. Currently, TTF-1, detecting in the immunohistochemistry panel, acts as an important prognostic marker for diagnosis of lung adenocarcinoma, which was also proposed by IASLC/ATS/ERS [3, 7–9]. Winslow *et al*. found increase of TTF-1 expression revealed a better outcome while reduced expression would enhance the ability of tumor seeding and metastasis [10]. Previous studies have demonstrated that positive TTF-1 expression (TTF-1+) is a significant prognostic factor for better outcome in pulmonary adenocarcinoma [11–14]. And some studies also revealed the relationship between TTF-1 expression and gene mutations that a higher epidermal growth factor receptor (EGFR) mutation rate would be found in TTF-1 positive adenocarcinoma [15, 16]. However, the patients in their researches were commonly with advanced diseases and the chemotherapeutic effect between TTF-1 positive adenocarcinoma and TTF-1 negative adenocarcinoma, especially in early-stage lung adenocarcinoma is unknown. Thus, we undertook an investigation of TTF-1 expression in stage I adenocarcinoma and compared the chemotherapeutic effect between TTF-1 positive adenocarcinoma and TTF-1 negative adenocarcinoma in pathological stage IB patients.

## RESULTS

### Clinicopathologic characteristics

In total, 2813 stage I patients including 126 adenocarcinomas with negative TTF-1 expression and 2687 adenocarcinomas with positive TTF-1 expression were subjected to the study. There were 1527 (54.3%) men and 1286 (45.7%) women in the series, with the mean age of 60.7 years (from 24 to 85 years).

The comparative clinicopathologic parameters between adenocarcinomas with positive TTF-1 expression and adenocarcinomas with negative TTF-1 expression are shown in Table 1. Sex, age, smoking history, T stage, disease stage, surgical procedure, subtypes and the treatment of adjuvant chemotherapy were analyzed. The characteristics between the two groups were similar.

**Table 1:** Clinicopathologic characteristics

Variable	TTF-1− (*n* = 126)	TTF-1+ (*n* = 2687)	*P*
Sex			0.769
Male	70	1457	
Female	56	1230	
Age			0.513
<65	79	1761	
≥65	47	926	
Smoking history			0.436
Smoker	8	223	
Non-smoker	116	2464	
T stage			0.486
1a	69	1483	
1b	26	667	
2a	31	537	
Stage			0.238
IA	95	2151	
IB	31	536	
Surgical procedure			0.928
Lobectomy	109	2332	
Wedge resection	17	355	
Subtypes			0.521
AIS	3	57	
MIA	3	201	
Lepidic	4	197	
Acinar	36	1098	
Papillary	23	851	
Micropapillary	1	32	
Solid	19	151	
Invasive mucinous	35	94	
Others	2	6	
Adjuvant chemotherapy			0.384
Yes	26	2214	
No	100	473	

Abbreviations: TTF-1, thyroid transcription factor 1; AIS, adenocarcinoma *in situ*; MIA, minimally invasive adenocarcinoma.

As for the relationship between TTF-1 expression and subtypes, among adenocarcinomas with negative TTF-1 expression, the major subtype was acinar-predominant adenocarcinomas (28.6%), followed by invasive mucinous (27.8%) and papillary (18.3%) subtypes while acinar (40.9%), papillary (31.7%) and minimally invasive adenocarcinoma (7.5%) were in the majority among adenocarcinomas with TTF-1+ (Table 1).

### Survival analysis

Univariable analysis revealed that age, disease stage, tumor size, T stage and surgical procedure were all significant factors for relapse-free survival (RFS) and overall survival (OS) (Table 2) while age and surgical procedure were still significant factors for RFS and OS in multivariable analysis (Table 3). The status of TTF-1 expression was not a significant factor for RFS and OS.

**Table 2:** Univariable analyses for RFS and OS in our study

Variable	RFS			OS		
	HR	95% CI	*p*	HR	95% CI	*p*
Age, yrs	1.915	1.425 to 2.575	**<0.001**	2.181	1.432 to 3.321	**<0.001**
Sex	1.180	0.878 to 1.585	0.273	1.269	0.833 to 1.934	0.267
Smoking history	0.749	0.434 to 1.295	0.301	0.930	0.448 to 1.932	0.846
Stage	3.396	2.525 to 4.567	**<0.001**	2.520	1.643 to 3.866	**<0.001**
T-size, cm	2.515	1.951 to 3.241	**<0.001**	1.991	1.407 to 2.818	**<0.001**
T Stage	2.232	1.861 to 2.677	**<0.001**	1.910	1.481 to 2.462	**<0.001**
Subtypes	0.988	0.945 to 1.034	0.614	0.990	0.929 to 1.055	0.754
TTF-1, +/−	0.730	0.414 to 1.287	0.277	0.601	0.299 to 1.207	0.152
Surgical procedure	0.402	0.274 to 0.592	**<0.001**	0.282	0.170 to 0.467	**<0.001**
Adjuvant chemotherapy	2.373	1.743 to 3.231	**<0.001**	1.332	0.820 to 2.161	0.246

Abbreviations: RFS, relapse-free survival; OS, overall survival; HR, hazard ratio.

**Table 3:** Multivariable analyses for RFS and OS in our study

Variable	RFS			OS		
	HR	95% CI	*p*	HR	95% CI	*p*
Age, yrs	1.644	1.206 to 2.240	**0.002**	1.687	1.094 to 2.062	**0.018**
Stage	1.858	0.835 to 4.134	0.129	0.934	0.310 to 2.817	0.903
T-size, cm	2.006	1.384 to 2.908	**<0.001**	1.464	0.878 to 2.440	0.144
T Stage	1.139	0.653 to 1.984	0.647	1.723	0.803 to 3.697	0.162
Surgical procedure	0.377	0.252 to 0.563	**<0.001**	0.272	0.160 to 0.464	**<0.001**
Adjuvant chemotherapy	1.384	0.969 to 1.977	0.074			

Abbreviations: RFS, relapse-free survival; OS, overall survival; HR, hazard ratio.

**Figure 1:**
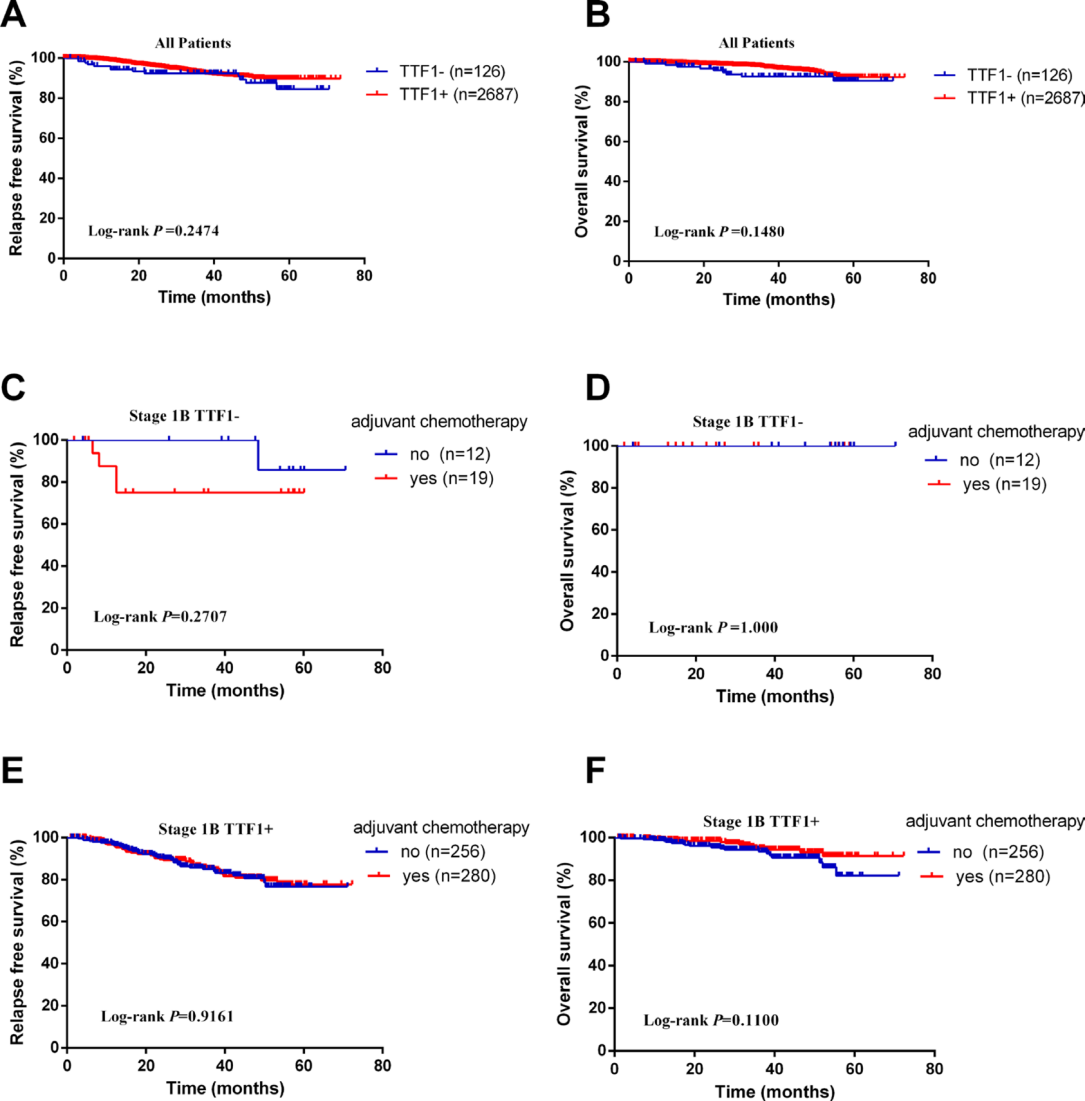
Kaplan–Meier survival curves for recurrence-free survival (RFS) and overall survival (OS) according to TTF-1 status (**A**) RFS in all patients. (**B**) OS in all patients. (**C**) RFS in stage IB TTF-1− patients. (**D**) OS in stage IB TTF-1− patients. (**E**) RFS in stage IB TTF-1+ patients. (**F**) OS in stage IB TTF-1+ patients.

In stage I adenocarcinomas, there was no survival difference between the two groups (RFS: Log-rank *p* = 0.2474; OS: Log-rank *p* = 0.1480, Figure 1A and 1B). When confined to stage IB adenocarcinomas with TTF-1−, whether received adjuvant chemotherapy made no difference to RFS and OS (RFS: Log-rank *p* = 0.2707; OS: Log-rank *p* = 1.000, Figure 1C and 1D), as was the case in stage IB adenocarcinomas with TTF-1+ (RFS: Log-rank *p* = 0.9161; OS: Log-rank *p* = 0.1100, Figure 1E and 1F).

**Figure 2:**
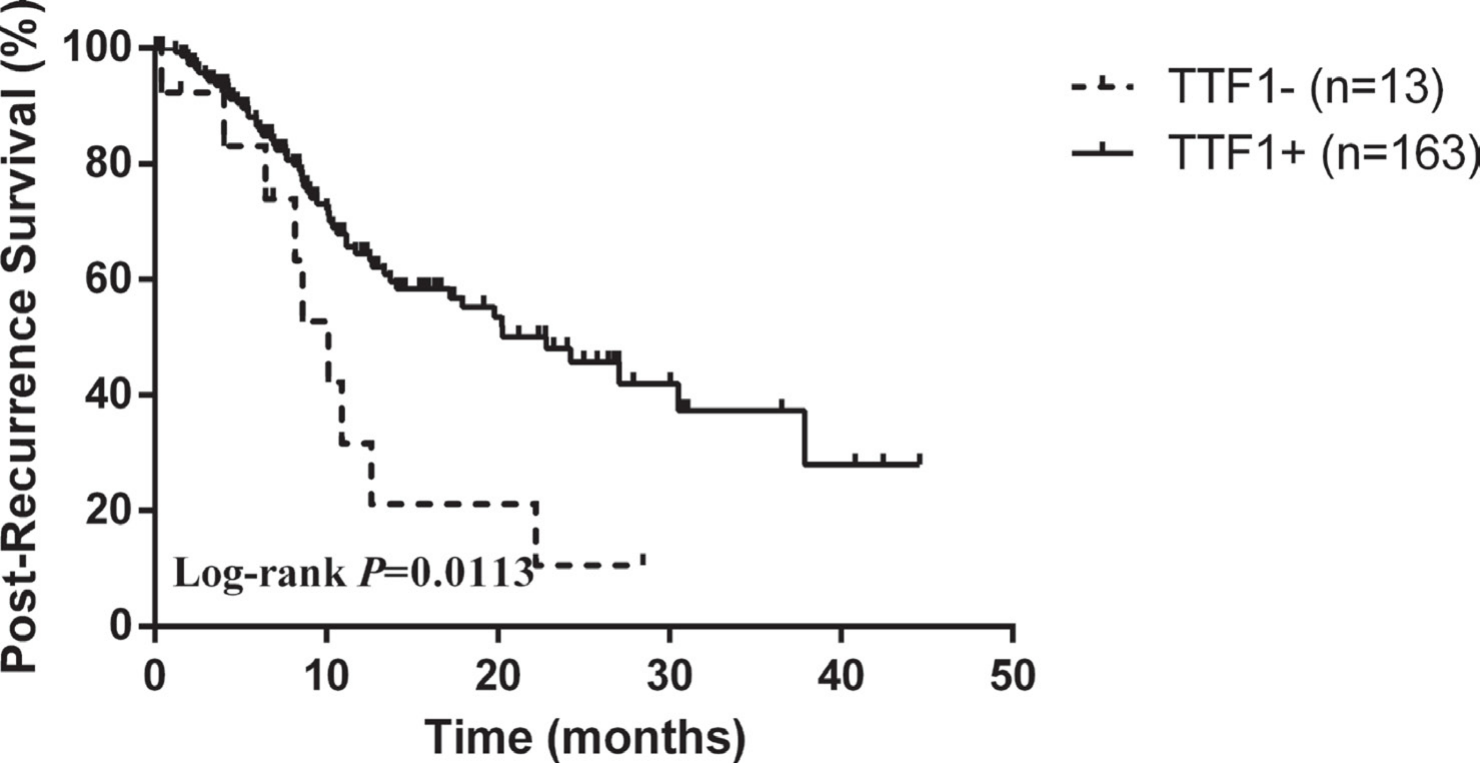
Kaplan–Meier survival curve for post-recurrence survival

Within follow-up period, a total of 13 (10.3%) TTF-1− patients and 163 (6.1%) TTF-1+ patients experienced a relapse. There was significant difference in post-recurrence survival (PRS) for TTF-1− patients compared with those TTF-1+ patients (Log-rank *p* = 0.0113, Figure 2). However, regarding to the recurrence site, there was no difference between TTF-1− patients and TTF-1+ patients in patients with stage I adenocarcinoma (30.8% versus 36.8%, *p* = 0.771).

## DISCUSSION

As IASLC/ATS/ERS emphasized in the new classification in 2011, immunohistochemical (including TTF-1), combined with histochemical and molecular studies were suggested to determine the specific type of lung cancer. Furthermore, TTF-1, acted as a pneumocyte marker, was considered to be the single best marker for lung adenocarcinoma and had the added value to distinguish primary lung cancer [3, 5, 17, 18]. In our study, we reviewed 2813 stage I patients including 126 adenocarcinomas with TTF-1+ and 2687 adenocarcinomas with TTF-1− to have a comprehensive understanding of the clinicopathologic features, patterns of recurrence, chemotherapeutic efficacy and the prognosis associated with TTF-1 expression. Zhang and his colleagues reported that patients with TTF-1− were inclined to be male smokers with larger tumor size and more advanced disease stage. Besides, patients with TTF-1− tended to have multiple metastases when they experienced a relapse [16]. Nevertheless, we found that in early stage adenocarcinomas, the characteristics between TTF-1+ group and TTF-1− group were similar, even the patterns of recurrence (*p* = 0.771). The reason may be because the more advanced disease stage was, the less TTF-1 would be expressed, and all the patients in our study were stage I patients while more patients with advanced stage in theirs.

In this study, TTF-1− adenocarcinomas most commonly appeared in acinar-predominant subtype, followed by followed by invasive mucinous and papillary subtypes, which was basically in accordance with previous studies [16, 19]. As Saad *et al* [20]. revealed, invasive mucinous adenocarcinoma was mostly negative for TTF-1, which was in consistence with our results. Interestingly, the 2011 IASLC/ATS/ERS classification indicated that all the nonmucinous adenocarcinoma *in situ* (AIS), minimally invasive adenocarcinoma (MIA) and lepidic adenocarcinoma were negative for TTF-1. But in our series, we found several patients were TTF-1+ (3 AISs, 3 MIAs, 4 lepidic adenocarcinomas), which needed further study.

Numerous studies have investigated the relationship between TTF-1 expression and prognosis, most of which demonstrated that positive TTF-1 expression was associated with better survival [11, 13, 21–23]. But in our study, there was no difference between TTF-1− group and TTF-1+ group in RFS (Log-rank *p* = 0.2474) and OS (Log-rank *p* = 0.1480), which was in accordance with Pelosi [24]. Moreover, we further investigated chemotherapeutic efficacy of stage IB adenocarcinomas between TTF-1− group and TTF-1+ group. Whether received adjuvant chemotherapy made no difference to RFS and OS (RFS: Log-rank *p* = 0.2707; OS: Log-rank *p* = 1.000, Figure 1C and 1D), as was the case in stage IB adenocarcinomas with TTF-1+ (RFS: Log-rank *p* = 0.9161; OS: Log-rank *p* = 0.1100, Figure 1E and 1F). The reason was as follows: it is generally known that on the promise of complete resection, the later pathological stage is, the poorer prognosis would be. All the patients in our study was stage I adenocarcinomas while more advanced disease made up the majority in other studies.

In our series, TTF-1 was not recognized as an independent prognostic factor, neither in univariable analysis nor in multivariable analysis. Disease stage may still be the major reason. Within the follow-up period, a total of 13 (10.3%) TTF-1− patients and 163 (6.1%) TTF-1+ patients experienced a relapse. There was significant difference in post-recurrence survival (PRS) for TTF-1− patients compared with those TTF-1+ patients (Log-rank *p* = 0.0113, Figure 2). The results was basically in line with zhang and his colleagues [16], who also found pathologic stage, TTF-1 expression and the use of EGFR TKIs as independent predictors of post-recurrence survival.

In recent years, molecular researches on mutational status were carried out widely. A higher EGFR mutation rate was identified in TTF-1+ adenocarcinomas, but a fraction of TTF-1− adenocarcinomas still had EGFR mutation [15, 25, 26]. They also revealed that under the treatment of EGFR TKIs, adenocarcinomas with TTF-1+ and EGFR mutation had a better outcome and in EGFR-mutated patients, those with TTF-1− adenocarcinoma had worse PFS than what those with TTF-1+ adenocarcinoma did [13]. This would be investigated in our future work.

There are several limitations in our study. First, this study is not a randomized prospective study, selection bias may exist. Second, in the subset of stage IB TTF-1− patients, the chemotherapeutic efficacy could not be shown well due to the small amount of sample size.

In summary, in patients with stage I adenocarcinoma, there is no significant difference in RFS and OS between TTF-1− group and TTF-1+ group, but TTF-1 negative adenocarcinoma has significantly worse PFS. Moreover, chemotherapeutic efficacy between TTF-1+ and TTF-1− stage IB adenocarcinomas did not differ.

## MATERIALS AND METHODS

From Jan. 2010 to Aug. 2015, we consecutively collected resected stage I pulmonary tumors in the Department of Thoracic Surgery, Shanghai Chest hospital. Two pathologists (J.K.Z and J.C.S.) confirmed the diagnosis by H&E staining and immunohistochemistry biomarkers. To exclude tumor metastasis, all the patients were under routine preoperative examinations including computed tomography (CT) or enhanced thoracic computed tomography, abdominal ultrasonography, brain magnetic resonance imaging (MRI) and bone scan. Furthermore, suspicious lymph node metastasis or distant metastasis which could not be excluded by routine preoperative examinations, endobronchial ultrasound-guided transbronchial needle aspiration (EBUS-TBNA) or positron emission tomography (PET)-CT scan was recommended. In our study, the inclusion criteria included:(1) patients with lung adenocarcinoma underwent R0 resection; (2) the status of TTF-1 (positive or negative) was available by immunohistochemistry test; (3) patients with complete follow-up records; (4) those with metastatic lung adenocarcinoma were excluded. Of these, we identified 2813 stage I adenocarcinomas with available clinicopathologic data and postoperative follow-up records.

Signed informed consents of patients were obtained and this study was approved by our institutional review board. All the corresponding clinicopathologic data including sex, age, smoking history, pathologic T stage, pathologic disease stage (according to AJCC TNM staging system 7th edition [17], surgical procedure, subtypes and adjuvant chemotherapy were investigated.

Immunohistochemical (IHC) staining was carried out on 4-μm formalin-fixed, paraffin-embedded tissue samples. Slides were deparaffinized and pretreated in 3% hydrogen peroxide and ethylenediaminetetraacetic acid successively. TTF-1 (1:100, 8G7G3/1, DAKO, glostrop, Denmark) was used at a 1:200 dilution. Then, the slides were washed in Tris-HCl and affected using horseradish peroxidase-conjugated anti-rabbit horseradish peroxidase-conjugated anti-rabbit EnVision+ kit (DAKO). Hematoxylin was applied to counterstain all the slides. Sole nuclear staining was considered TTF-1 positive.

### Statistical analysis

SPSS 19.0 software package (SPSS Inc, Chicago, IL) was used to analyze the clinicopathologic data and distributions of relapse-free survival (RFS), overall survival (OS) and post-recurrence survival (PRS) were established by Prism 5 (Graph Pad Software Inc., La Jolla, CA), using the Kaplan–Meier method, and the comparisons between two categories was employed by the log-rank test. A *p* value less than 0.05 was considered as statistically significant.

Cox regression was adopted to perform univariable and multivariable analyses. The end point was recognized as event occurrence (relapse for RFS while death for OS). All the correlative clinicopathologic characteristics were enrolled into univariable analysis one by one, with the method “Enter”. Moreover, all the correlative clinicopathologic characteristics were brought into multivariable analysis together when *p* value had significance (<0.05) in univariable analysis.

## Abbreviations

TTF-1: thyroid transcription factor-1
RFS: relapse-free survival
OS: overall survival
PRS: post-recurrence survival
IASLC: the International Association for the Study of Lung Cancer
ATS: American Thoracic Society
ERS: European Respiratory Society
EGFR: epidermal growth factor receptor
AIS: adenocarcinoma *in situ*
MIA: minimally invasive adenocarcinoma
CT: computed tomography
MRI: magnetic resonance imaging
IHC: Immunohistochemical
EBUS-TBNA: endobronchial ultrasound-guided transbronchial needle aspiration
PET: positron emission tomography
